# Molecular basis of the anti-hyperglycemic activity of RA-3 in hyperlipidemic and streptozotocin-induced type 2 diabetes in rats

**DOI:** 10.1186/s13098-019-0424-z

**Published:** 2019-03-29

**Authors:** Sihle Ephraim Mabhida, Rabia Johnson, Musawenkosi Ndlovu, Johan Louw, Andrew Opoku, Rebamang Anthony Mosa

**Affiliations:** 1 Department of Biochemistry and Microbiology, University of Zululand, Private Bag X1001, KwaDlangezwa, 3886 South Africa; 2 Biomedical Research and Innovation Platform (BRIP), South African Medical Research Council, Tygerberg, 7505 South Africa; 3 Division of Medical Physiology, Tygerberg, Stellenbosch University, Stellenbosch, South Africa

**Keywords:** Streptozotocin, Hyperglycemia, Lanosteryl triterpene, High-fat diet, *Protorhus longifolia*

## Abstract

**Background:**

Insulin resistance is a hallmark of type 2 diabetes mellitus (T2DM) and the underlying cause of various metabolic changes observed in type 2 diabetic patients. This study investigated the molecular basis of the anti-hyperglycemic activity of the lanosteryl triterpene (RA-3), from *Protorhus longifolia* stem bark, in hyperlipidemic and streptozotocin (STZ)-induced T2DM in rats.

**Methods:**

The high-fat diet fed (HFD) and STZ-induced T2DM in rat model was used to evaluate the anti-hyperglycemic activity of RA-3. The hyperlipidemic rats received a single intraperitoneal injection of STZ (35 mg/kg body weight) to induce T2DM. The experimental animals received a daily oral single dose of RA-3 (100 mg/kg body) for a period of 28 days, whiles the control group received distilled water only. The animals were euthanized, and skeletal muscle was collected for protein (IRS-1, AKT, GSK and GLUT 4) expression analysis. Western blot confirmed expression of the proteins.

**Results:**

Treatment of the diabetic animals with the RA-3 showed marked reduction in fasting plasma glucose levels in comparison to the untreated diabetic group animals. A significant decrease in p-GSK-3β and p-AKT expression was observed, whereas the expression of IRS-1^ser307^ were increased when compared to the diabetic control group. This effect was ablated upon treatment with RA-3 and this was concomitant to an observed increase in GLUT 4 expression.

**Conclusions:**

The results obtained in the present study strongly suggested that the anti-hyperglycemic effect of RA-3 could partly be associated with its ability to improve cellular glucose uptake in muscle tissue from T2DM.

## Introduction

The use of medicinal plants and their derived bioactive compounds to cure various ailments, including metabolic disorders, has been a preference since the earliest of times. There is still a continuous rising interest in the use of medicinal plants either in their crude or pure form to fight diseases such as diabetes mellitus (DM). Triterpenes, a unique class of plant-derived chemicals containing three terpene units, have recently gained much interest as new targets towards development of new pharmacologically active drugs. This is due to their wide range of significant biological activities including anti-diabetic properties [1–3]. Their hypoglycemic effect has been associated with their ability to decrease intestinal glucose absorption [4] and stimulate insulin secretion and cellular glucose uptake in peripheral tissues [5]. Several in vitro and in vivo studies have demonstrated the potential of natural triterpenoids to enhance the insulin signaling pathway and protect and regenerate pancreatic islets [2, 6, 7]. This class of compounds is also known for its inhibitory activity against alpha glucosidase and alpha amylase [8], inhibition of protein tyrosine phosphatase 1B and inhibition of glycogen phosphorylase [9]. Triterpenes have also demonstrated a strong potential for the treatment of DM associated complications such as cardiomyopathy [10] and beta cell dysfunction [3].

Experimental data in our laboratory have demonstrated that RA-3, a lanosteryl triterpene from *Protorhus longifolia* (Benrh.) Engl. (Anacardiaceae) stem bark, possesses cardioprotective effect [11], anti-hyperlipidaemic [12], and anti-hyperglycaemic activities [13]. This compound has been reported to improve glycemic control and pancreatic beta cell ultrastructure in HFD-STZ-induced type 2 diabetic animals [14]. However, the molecular mechanism(s) through which the compound improves the glucose tolerance in the type 2 diabetic animals remains to be explored. Therefore, this study is aimed at evaluating the molecular mechanism by which RA-3 improves glucose tolerance in the hyperlipidemic and STZ-induced type 2 diabetic rats.

## Materials and methods

### The lanosteryl triterpene extraction and isolation

The fresh stem bark of *Protorhus longifolia* (specimen voucher number RAUZ01) was collected from KwaZulu-Natal, SA and routinely prepared for extraction. The isolation of the RA-3 was performed from the chloroform extract of the powered stem bark of *Protorhus longifolia* using chromatographic techniques as previously reported [11–14].

### Animals

*Sprague*–*Dawely* rats (150–200 g) of either sex were provided by the departmental animal unit of Biochemistry and Microbiology, University of Zululand. Rats were housed and maintained under standard conditions [12-h light/dark cycle, humidity (~ 50%), temperature (23–25 °C)]. Before commencement of experimental procedures, the rats were acclimatized for a period of 5 days with free access to water and pelleted rat feed. The University of Zululand Research Ethics Committee (UZREC) granted the approval for procedures and use of laboratory animals (*Rattus norvegicus*) (UZREC 171110–030 PGM 2016/329).

### Induction of hyperlipidemia

The induction method of hyperlipidemia in rats was performed with some modification as previously described by Machaba et al. [12, 14]. The animals in the experimental group were placed on HFD for 28 days. The blood cholesterol levels were measured using ACCUTREND PLUS^®^ cholesterol strips (Roche Products) from the rat’s tail tip to confirm the hyperlipidemic state. Blood cholesterol levels above or equal to 5.2 mmol/L were used to confirm the hyperlipidemic state of the animals to be used in the study.

### Type 2 diabetes induction

The hyperlipidemic rats were fasted overnight and then intraperitoneally injected with a freshly prepared low dose of STZ solution (35 mg/kg) to induce diabetes (T2DM) [15, 16]. The blood glucose levels were measured after 5 days using a glucometer (ACCUTREND PLUS^® glucose^ strips, Roche Products) from the rat’s tail tip to confirm the hyperglycemic state of the animals. Blood glucose levels above or equal to 11 mmol/L were used to confirm the diabetic state of the animals to be used in the study [17].

### In vivo anti-hyperglycemic activity

The hypoglycemic activity of RA-3 was assessed in the type 2 diabetic animals following the method done by Kumar et al. [14, 18]. Animals were randomized into four groups (n = 5), fed either a standard rodent diet, while the HFD-diabetic (HFD-D) groups received either 2% Tween 20 (vehicle control), a single dose of RA-3 (100 mg/kg) or metformin (100 mg/kg) orally for 28 days. The non-diabetic group received equivalent amount of distilled water. The animals were allowed free access to water and pelleted rat feed throughout the experimental period. Blood glucose levels were determined weekly. After 28 days, animals were overnight fasted and euthanized and skeletal muscles tissue harvested for western blot analysis.

### Western blot analysis of some proteins involved in insulin signaling pathway

The frozen skeletal muscle was weighed, defrosted on ice and lysed in lysis buffer (800 μL) (Pierce Biotechnologies, Rockford, CA, USA) using a tissue lyser at 25 Hz. Thereafter, the samples were centrifuged for 20 min at 12,000 rpm at 4 °C in a micro centrifuge. The supernatant was collected and kept in a fresh tube at − 20 °C until required. Protein (30 µg) was mixed with an equal volume of 2× Laemmli sample buffer (containing β-mercaptoethanol), before it was denatured at 95 °C. The denatured protein sample (30 µg) was loaded onto a 12% Mini-protein TGX Precast Gels, 10 well comb, 50 µL/well (BioRad), along with 12 µL BioRad-Western C molecular weight marker. The gel was run for 1 h at 150 V in Tris/Glycine/SDS-PAGE Buffer 1× and then transferred to a polyvinylidene fluoride membrane (PVDF, Bio-Rad, Hercules, CA, USA) [19]. Membranes were incubated at 4 °C for 16 h with different primary antibodies which include IRS-1^Ser307^, p-AKT and p-GSK-β, [Cell Signaling Technology, Danvers, MA, USA; (1:500), (1:1000), (1:1000) respectively], GLUT 4 [Sigma-Aldrich Chemical Co., St. Louis, MO, USA, (1:1000)]. β-Actin [Cell Signaling Technology, Danvers, MA, USA, (1:4000)] antibody was added as a loading control. The membrane was incubated with an appropriate horseradish peroxidase conjugated secondary antibody (1:4000) in blocking buffer (2.5% milk) at room temperature for 90 min, and then placed on a plastic sheet and 2 ml chemiluminescent substrate (1:1 of luminal solution and reaction buffer) was added to the membrane for signal development. The proteins were detected using BioRad ChemiDoc and the signaling intensity of the bands was quantified using the image J software.

## Results

### RA-3 effect on blood glucose in the HFD-STZ-induced type 2 diabetic animals

**Fig. 1:**
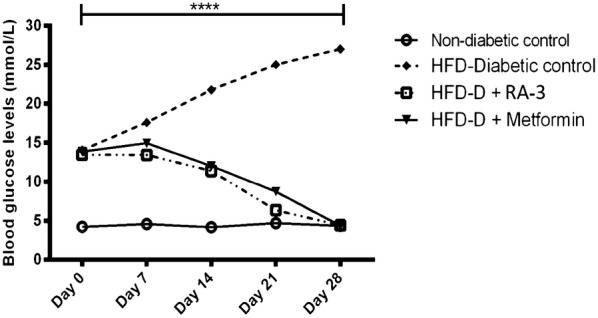
The activity of RA-3 on FBG after the 28 days treatment of the type 2 diabetic animals. Values are expressed as the mean ± SEM (*n *= 5). ***p ≤ 0.001, vs. non-diabetic control. *D* diabetic

Figure 1 shows the biological activity of RA-3 on fasting blood glucose (FBG) levels of the type 2 diabetic rats. While a persistently higher fasting plasma level of glucose was observed in the diabetic control group, treatment of the diabetic animals with RA-3 effectively lowered their blood glucose levels. The observed effects were similar and comparable to those of the metformin treated group.

### Effect of RA-3 on some insulin signaling proteins (p-GSK-3β, p-AKT, IRS-1^ser307^ and GLUT 4)

**Fig. 2:**
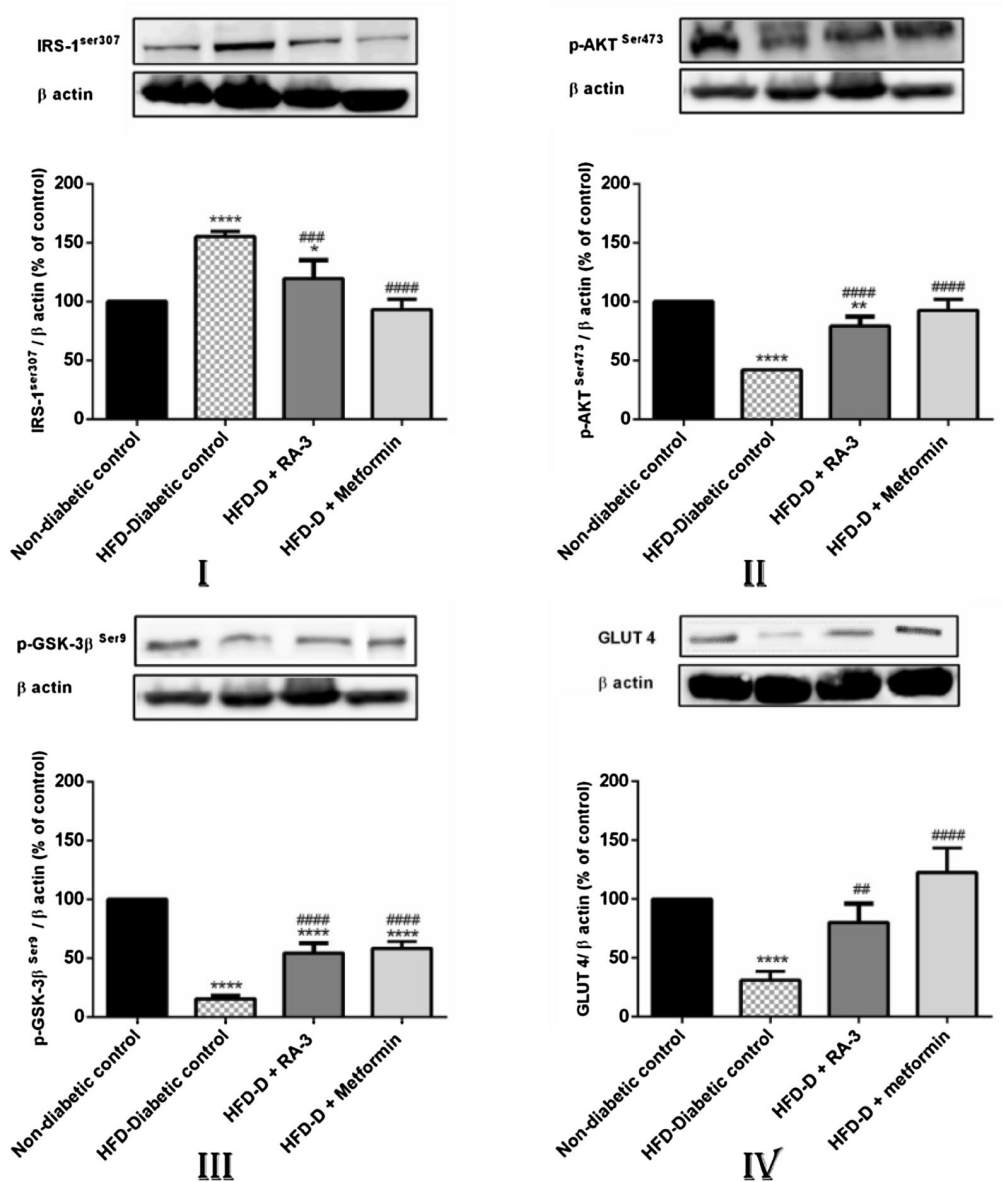
Activity of RA-3 on IRS-1^ser307^ (**I**), p-AKT ^Ser473^ (**II**), p-GSK-3β ^Ser9^ (**III**) and GLUT 4 (**IV**) protein expression in type 2 diabetic rats. Values are expressed as the mean ± SEM (*n *= 5). *p ≤ 0.05, **p ≤ 0.01, ****p ≤ 0.0001 vs. non-diabetic control, ^###^p ≤ 0.001, ^####^p ≤ 0.0001 vs. diabetic control. *D* diabetic

Figure 2 shows the findings of the activity of RA-3 on some insulin signaling proteins. A relatively low expression of p-GSK-3β, p-AKT and GLUT 4 along with higher expression of IRS-1^ser307^ was observed in the diabetic control group vs non-diabetic control group. However, following treatment of the diabetic animals with RA-3, higher expression of p-GSK-3β, p-AKT and GLUT 4 and lower expression of IRS-1^ser307^ were observed in comparison with the untreated diabetic group. The observed effects were similar and comparable to the metformin treated group.

## Discussion

Experimental evidence has shown that oxidative stress and increase in pro-inflammatory cytokines is closely related to insulin resistance, through their negative activity on insulin signaling pathways and glucose transport into the cell. Thus, their effects have been suggested to explain the development of insulin resistance in T2DM [20]. RA-3 has recently established to improve glycaemic control and pancreatic beta cell ultrastructure in HFD-STZ-induced type 2 diabetic rats. The pancreatic beta cells protective effect of RA-3 has been linked to the ability to enhance antioxidant status and reduce the release of pro-inflammatory cytokines such as IL-6 [14]. However, the molecular mechanism(s) through which the compound improves the glycaemic control in the type 2 diabetic animals remained to be explored [14]. Thus, the current study aimed at evaluating the molecular mechanism through which RA-3 improves glycaemic control in the hyperlipidemic and STZ-induced type 2 diabetic animals. Normally insulin stimulates cellular glucose uptake by activating a cascade of reactions leading to plasma membrane recruitment of glucose transporters GLUTs. The translocation of these GLUTs is regulated by a series of proteins such as PI3-K, AKT and IRS-1. However, while PI3-K and AKT are known to be downregulated in T2DM, IRS-1^Ser307^ is known to be highly expressed inhibiting insulin signalling pathway and glucose uptake [21].

Interestingly, the results from this study demonstrated that RA-3 was able to stimulate the insulin signaling pathway by reducing the expression of IRS-1^Ser307^ and increased p-AKT/p-GSK-3β expression after 28 days of treatment. These findings were further evidenced by the increased GLUT 4 expression and the subsequent anti-hyperglycemic effect indicated by an effective reduction of the FBG levels. Phosphorylated GSK-3β at ser9 by AKT is well-known to play a major role in insulin-induced stimulation of glycogen synthesis. The ability of RA-3 to increase the expression of p-GSK-3β shows the potential of this compound to control glucose homeostasis by maintaining the balance between glucose storage and glycogen breakdown, while preventing chronic gluconeogenesis. The results obtained from this study provides the molecular basis through which RA-3 improves glucose tolerance in HFD-STZ-induced type 2 diabetic animals, which has recently been established in our laboratory [14]. The results are also similar and consistent with those observed in the type 1 diabetes model reported by Mabhida et al. [22]. The results from this study could also partly serve to explain the stimulatory effect of RA-3 on glucose uptake by C2C12 myocytes and 3T3-L1 adipocytes previously reported by Mosa et al. [23]. Oleanolic acid has also been reported to exert its hypoglycemic effects through activation of the insulin signaling pathway in skeletal muscle of STZ-induced diabetic rats [2].

## Conclusion

The findings obtained in the present study confirm that the hypoglycemic activity of RA-3 is through its apparent ability to enhance the insulin signaling pathway and consequently increase the expression of GLUTs in the skeletal muscles of diabetic animals. These findings suggest that RA-3 could be a potent therapeutic candidate in the development of new anti-diabetic drugs. However, further molecular studies in which RA-3 regulates the functioning of pancreatic beta cells and other related organs are still required.

## Abbreviations

T2DM: type 2 diabetes mellitus
RA-3: lanosteryl triterpene
STZ: streptozotocin
HFD: high-fat diet fed
BGL: blood glucose levels
GLUT 4: glucose transporter 4
GSK-3β: glycogen synthase kinase 3 beta
IRS-1: insulin receptor substrate
AKT: protein kinase B
